# Gastrointestinal parasites of canids, a latent risk to human health in Tunisia

**DOI:** 10.1186/s13071-017-2208-3

**Published:** 2017-06-05

**Authors:** Myriam Oudni-M’rad, Raja Chaâbane-Banaoues, Selim M’rad, Fatma Trifa, Habib Mezhoud, Hamouda Babba

**Affiliations:** 1 Laboratory of Medical and Molecular Parasitology-Mycology (LP3M), LR 12ES08. Faculty of Pharmacy, University of Monastir, 5000 Monastir, Tunisia; 2 Laboratory of Biomass Valorisation and Production of Eukaryotic Proteins, Center of Biotechnology, University of Sfax, 3018 Sfax, Tunisia; 3 Laboratory of Parasitology-Mycology, EPS F. Bourguiba, 5000 Monastir, Tunisia

**Keywords:** Gastrointestinal parasites, Environmental parasite contamination, Zoonosis, Tunisia, Canids

## Abstract

**Background:**

Although data on the parasite environmental contamination are crucial to implement strategies for control and treatment, information about zoonotic helminths is very limited in Tunisia. Contamination of areas with canid faeces harboring infective parasite elements represents a relevant health-risk impact for humans. The aim of this study was to assess the environmental contamination with eggs and oocysts of gastrointestinal parasites of dogs and wild canids in Tunisia with special attention to those that can be transmitted to humans.

**Results:**

One thousand two hundred and seventy faecal samples from stray dogs and 104 from wild canids (red foxes and golden jackals) were collected from different geographical regions throughout Tunisia. The helminth eggs and protozoan oocysts were concentrated by sucrose flotation and identified by microscopic examination. The most frequently observed parasites in dog samples were *Toxocara* spp. (27.2%), *E. granulosus* (25.8%), and Coccidia (13.1%). For wild canid faeces, the most commonly encountered parasites were *Toxocara* spp. (16.3%) followed by *Capillaria* spp. (9.6%). The parasite contamination of dog faeces varied significantly from one region to another in function of the climate.

**Conclusion:**

To our knowledge, the study highlights for the first time in Tunisia a serious environmental contamination by numerous parasitic stages infective to humans. Efforts should be made to increase the awareness of the contamination risk of such parasites in the environment and implement a targeted educational program.

## Background

Parasitic infections are among the most common infections worldwide, principally in developing countries with poor environmental sanitation and inadequate personal hygiene. In particular, soil-transmitted helminths (*Ascaris lumbricoides*, whipworm (*Trichuris trichiura*) and hookworm) causing the “neglected infections of poverty” have a relevant health-risk impact on humans and animals [1–5]. Nevertheless, gastrointestinal parasites of canids constitute another source of human and livestock infection mainly due to environmental contamination with faeces containing infective parasitic forms (eggs, larvae or oocysts) [6–8]. Several serious parasitic diseases transmitted by dogs, such as hydatidosis and toxocariasis, are regarded as serious public health problems especially in Mediterranean countries [9–11]. Wild canids are also the reservoir of a wide range of parasites including parasites that are shared between pets and humans [12–14]. Human infections are acquired *via* the ingestion of eggs or oocysts via contaminated foodstuffs or water, hands, inhalation of dust, and/or by penetration of larvae through the skin [15]. Geophagia (eating of earth) and shoeless walking are the most common risk factors of contamination in children [16, 17].

In Tunisia, the canine population is estimated at 800,000 dogs and is essentially composed of stray and semi-stray (free-roaming dogs which are fed by an owner) dogs that rarely receive deworming treatment [18]. The uncontrolled displacement of canids in rural and urban areas increases the contamination risk of the soil, food and water with parasitic elements. The dog faeces are not removed from the ground and may be a serious hazard for human health. Epidemiological studies have been performed on gastrointestinal helminths in necropsied dogs [19–21] and wild canids [21, 22] in Tunisia but relatively little information on the environmental contamination by protozoan oocysts or helminth eggs is currently available [23]. Thus, understanding the epidemiology of zoonotic parasitic infections due to canids is necessary to minimize the exposure risk to humans.

The aim of the present work was (i) to assess the data from an epidemiological survey of environmental contamination with helminth eggs and protozoan cysts of dogs, with special attention to those that can be transmitted to humans; and (ii) to investigate the possible role of wild canids in the transmission of gastrointestinal parasites to humans.

## Methods

### Sample collection

**Fig. 1:**
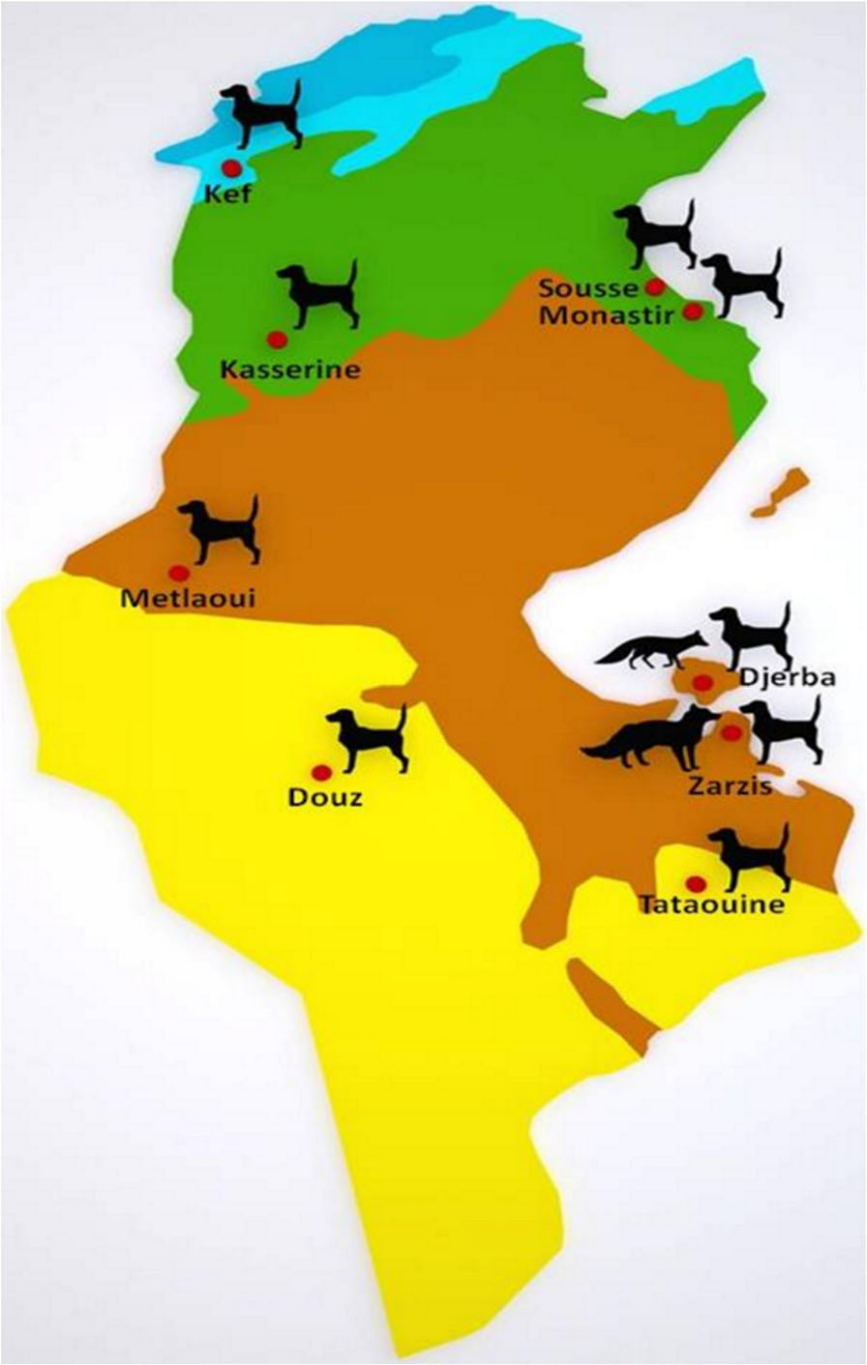
Faecal sampling collections from dogs and wild canids in different Tunisian locations according to the climate: humid (*blue*), sub-humid (*turquoise*), semi-arid (*green*), Arid (*brown*), desertic (*yellow*)

One thousand two hundred and seventy faecal samples from dogs (*Canis familiaris*) were collected from the soil from four climate zones in Tunisia: sub-humid (Kef), semi-arid (Kasserine, Sousse and Monastir), arid (Djerba Island, Zarzis and Metlaoui), and desertic (Douz and Tataouine) (Fig. 1). Forty samples were randomly collected from a proportion of the faeces observed over a soil surface of 200–400 m^2^ according to the abundance of faeces in each location. One hundred and four faeces from wild canids were isolated: 88 faecal samples around red fox burrows (*Vulpes vulpes*) from Djerba Island and 16 faeces from the rest sites of golden jackals (*Canis aureus*) from Zarzis. The samples were collected with the help of hunters and experienced forestry technicians based essentially on the defecation sites, the shape and size of faeces, and the footprints left by wild canids. All faeces were taken from rural, semi-urban, and urban sites in the vicinity of livestock breeding (ovine and bovine). Houses and animal husbandries were observed around all the study areas.

Several sites were visited for each region (2–5) and samples were collected in spring and summer. The sampling was not related to a number of individual canids, but intended to represent the available parasite eggs or oocysts in the area. Faecal samples were collected without alcohol or formalin fixation and frozen at -80 °C for 7 days in order to partially inactivate infective stages of the parasites.

### Microscopic and molecular analysis

The gastrointestinal helminth eggs and protozoan oocysts were concentrated by sucrose density gradient flotation with a specific gravity of 1.27 [24]. Slides were then systematically microscopically checked at 40× magnification. Using morphological and morphometric characteristics, each egg or protozoan oocyst was identified by light microscopic examination [25]. With the exception of *Echinococcus granulosus* and *Dipylidium caninum*, isolated parasites were identified at the family/genus level. Since the taeniid eggs are morphologically indistinguishable, Eg1121/1122 PCR was used to identify the *E. granulosus* among taeniid egg-positive samples. Briefly, an alkaline lysis using DTT (dithiothreitol) and KOH (potassium hydroxide) followed by an enzymatic digestion by proteinase K (Invitrogene, Karlsruhe, Germany) were performed to destroy the embryophore’s rigid shell. The total DNA was extracted using a phenol chloroform protocol [26]. The PCR approach was realized as previously described by Chaabane-Banaoues et al. [23].

The percentage of faecal samples found positive for at least one parasitic element (egg or oocyst) provided the parasite contamination index which was estimated as the number of positive parasite isolates/total number of examined samples in each location.

### Statistical analysis

The categorical variables were expressed as percentages and the exact binomial confidence intervals were calculated (95% CI). The Pearson’s Chi-square and the Spearman’s correlation tests were used to compare the contamination indices according to the host (wild or domestic canid), the sampling regions, and the relationship between parasites (SPSS software, version 18.0). All *P*-values less than 0.05 were considered as statistically significant.

The dog faeces contamination indices higher than 4%, the temperature (annual average maximum temperature), and the rainfall (average yearly rainfall) of studied regions were compared using the principal component analysis (PCA) with MVSP software (Multivariate statistical package. MVSP. User’ manual. Version 3.1. KCS, 288. Pentraeth, Wales, UK. 2002).

## Results

**Fig. 2:**
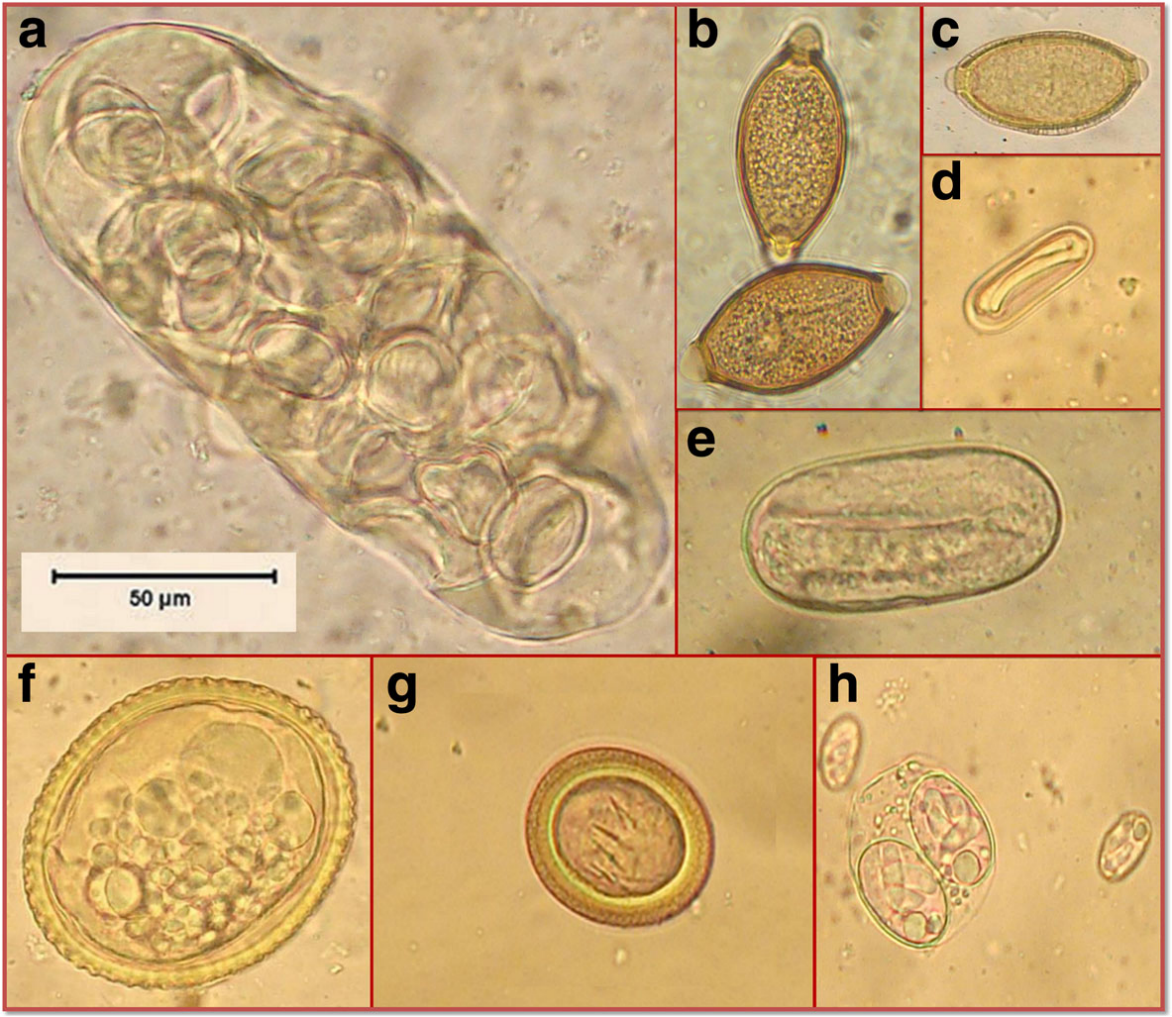
Cestode and nematode eggs and oocysts observed in the canid faecal samples. **a** *D. caninum* egg-capsule. **b** *Trichuris* spp. **c** *Capillaria* spp. **d** *Spirocerca* spp. **e** *Ancylostoma* spp. **f** *Toxocara* spp. **g** Taeniidae. **h** Coccidian oocysts

The present study revealed the presence of numerous pathogenic helminth eggs and protozoan oocysts (Table 1 and Fig. 2). The overall contamination index was 55% (95% CI: 52.2–57.7) and 46.1% (95% CI: 36.5–55.6) for dog and wild canid faecal samples respectively. Multiple infections were less frequent (30.1%, 95% CI: 26.8–33.3) than single infection (70%, 95% CI: 66.7–73.2) in dog faeces (from all regions). Similar results were obtained for the wild canid (Djerba-Zarzis) samples, and 38.5% (95% CI: 29.1–47.8) of them presented multiple infections versus 61.5% (95% CI: 52.2–70.9) for single infections (data not shown).

**Table 1:** Parasite contamination index of dog faeces in relation to regions and climate

Climate	Sub-humid	Semi-arid			Arid			Desertic			Overall contamination index
Locality	Kef (*N* = 36)	Kasserine (*N* = 132)	Monastir (*N* = 95)	Sousse (*N* = 81)	Metlaoui (*N* = 392)	Zarzis (*N* = 129)	Djerba (*N* = 127)	Tataouine (*N* = 103)	Douz (*N* = 175)	*P*-value	
Parasite	% (*n*)	% (*n*)	% (*n*)	% (*n*)	% (*n*)	% (*n*)	% (*n*)	% (*n*)	% (*n*)		
*Toxocara* spp.	16.7 (6)	17.4 (23)	30.5 (29)	17.3 (14)	29.3 (115)	17.8 (23)	51.2 (65)	21.3 (22)	26.8 (47)	<0.00001	27.2 (344)
Taeniidae	8.3 (3)	18.9 (25)	10.5 (10)	13.6 (11)	44.6 (175)	17.8 (23)	29.1 (37)	14.6 (15)	28.5 (50)	<0.00001	27.4 (349)
*E. granulosus* ^a^	8.3 (3)	18.2 (24)	9.6 (9)	12.3 (10)	41.3 (162)	17.8 (23)	27.6 (35)	14.6 (15)	26.8 (47)	<0.00001	25.8 (328)
*Trichuris* spp.	52.8 (19)	0.7 (1)	20 (19)	16 (13)	0 (0)	2.3 (3)	3.1 (4)	0 (0)	1.1 (2)	<0.00001	4.8 (61)
*Ancylostoma* spp.	5.5 (2)	6.8 (9)	4.2 (4)	8.6 (7)	3.7 (14)	5.4 (7)	5.5 (7)	8.7 (9)	0 (0)	0.0193	4.6 (59)
*Capillaria* spp.	0 (0)	0 (0)	0 (0)	0 (0)	0 (0)	0.8 (1)	0 (0)	0 (0)	0 (0)	0.3549	0.1 (1)
*Spirocerca* spp.	5.5 (2)	0 (0)	1 (1)	0 (0)	0 (0)	0 (0)	0 (0)	0 (0)	0 (0)	0.0013	0.2 (3)
*D. caninum*	0 (0)	0.7 (1)	0 (0)	0 (0)	0 (0)	0 (0)	0 (0)	0 (0)	0 (0)	0.3746	0.1 (1)
Coccidia	38.9 (14)	24.2 (32)	26.3 (25)	4.9 (4)	15.5 (61)	10.1 (13)	8.7 (11)	0.9 (1)	2.8 (5)	<0.00001	13.1 (166)

^a^ *E. granulosus* eggs were identified on biomolecular basis

*Abbreviations*: *N* Number of faecal samples analysed, *n* Number of positive samples, *%* Percentage of positive samples

Parasite distribution was significantly different between the dogs and wild canids in Zarzis and Djerba for *E. granulosus (χ* ^2^ = 28.95, *df* = 1, *P* < 0.0001), *Toxocara* spp. (*χ* ^2^ = 11.63, *df* = 1, *P* = 0.0006), *Capillaria* spp. (*χ* ^2^ = 21.25, *df* = 1, *P* < 0.0001) and *Spirocerca* spp.(*χ* ^2^ = 4.95, *df* = 1, *P* = 0.026) (Table 2). For the dog faeces, the most frequently observed parasites were *Toxocara* spp. (27.2%), *E. granulosus* (25.8%) and Coccidia (13.1%) (Table 1). Thanks to biomolecular analysis, 94% of Taeniidae samples (328 faeces) were identified as *E. granulosus* eggs (Table 1). Nevertheless, it should be noted that co-infection with other species of Taeniidae cannot be excluded. The wild canids were predominately infected with *Toxocara* spp. (16.3%) and *Capillaria* spp. (9.6%) (Table 2). No *E. granulosus* egg was detected in wild canids in Djerba and Zarzis regions whereas dog faeces were largely contamined (22.6%) (Table 2).

**Table 2:** Intestinal parasites in canids based on eggs and oocysts recovered in the faeces from Djerba-Zarzis regions

	Dogs (*N* = 256)	Wild canids (*N* = 104)	Dogs *vs* wild canids	
	% (*n*)	% (*n*)	Chi-square test	*P*-value
*Toxocara* spp.	34.3 (88)	16.3 (17)	11.63	0.0006
Taeniidae	23.4 (60)	0 (0)	29.25	< 0.0001
*E. granulosus* ^a^	22.6 (58)	0 (0)	28.95	< 0.0001
*Ancylostoma* spp.	5.4 (14)	3.8 (4)	0.41	0.5219
*Trichuris* spp.	2.7 (7)	6.3 (7)	3.16	0.0754
*Capillaria* spp.	0.4 (1)	9.6 (10)	21.25	< 0.0001
*Spirocerca* spp.	0 (0)	1.9 (2)	4.95	0.0260
Coccidia	9.3 (24)	7.7 (8)	0.25	0.6108

^a^ *E. granulosus* eggs were identified on biomolecular basis

*Abbreviations*: *N* Number of faecal samples analysed, *n* Number of positive samples, *%* Percentage of positive samples

The parasite environmental contamination varied significantly from one region to another (Table 1). *Echinococcus granulosus* was predominant for the faecal samples from the arid region whereas *Trichuris* spp. was the most frequently observed parasite for the sub-humid area (Table 1). The other regions had a similar parasite distribution and *Toxocara* spp. was the most commonly parasite found. *Dipylidium caninum*, *Spirocerca* spp. and *Capillaria* spp. were present in dog faeces with an occurrence lower than 1% (Table 1).

**Fig. 3:**
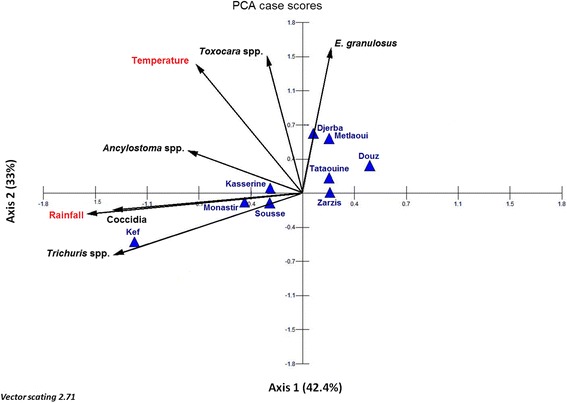
Parasite contamination indices and Tunisian region bioclimatic characteristics described by principal component analysis. Temperature (C°): annual average maximum temperature; Rainfall (mm): annual average rainfall

As for the dog samples, the Spearman’s correlation coefficient demonstrated a positive correlation between *E. granulosus* and *Toxocara* spp. eggs (*r*_*s*_ = 0.55, *P* = 0.132), and a negative correlation between *E. granulosus* and *Trichuris* spp. eggs (*r*_*s*_ = -0.67, *P* = 0.044). The PCA graphics highlighted that *Trichuris* spp*., Ancylostoma* spp., and *Coccidia* distributions were significantly and positively correlated with rainfall (Fig. 3, Axis 2)*.* The *E. granulosus* and *Toxocara* spp. isolates were ubiquitous even in high temperature regions (Fig. 3, Axis 1).

## Discussion

Although data on the regional prevalence of parasites are crucial to implement strategies for control and treatment, information about parasite environmental contamination is very limited in Tunisia. Except for cystic echinococcosis, epidemiological studies of zoonotic helminths are rare and only few human case reports have been published. Significant levels of parasitism were observed more in dogs (55%) than in wild canids (46.1%). These contamination levels are comparable to those described for dog faecal samples in other countries such as Cuba (44.3%) [27], Canada (33.9%) [28] and Portugal (59.8%) [29]. To our knowledge, we cannot exclude that the faeces contamination indices were overestimated due to the coprophagic behaviour of canids (consummation of their own faeces, faeces of other canids and/or faeces of other species) [30]. The zoonotic agents *Toxocara* spp., *E. granulosus*, *Ancylostoma* spp. and *Trichuris* spp. were the most frequent parasites observed in the study. The situation presented here is grossly the same as that in several parts of the world such as Argentina [31], North America [32] or Iran [33]. Helminths encountered in the present study have at least one form infective to humans: the eggs for orally-ingested parasite species, and the free-living stages for skin-penetrating species. The faecal-oral route is the most common way of helminth and protozoan contamination. Thus, the consumption of uncooked vegetables irrigated by water polluted by animal faeces and/or soil ingestion could have a direct impact on human health and cause severe zoonoses [34, 35].

Contrary to previous studies that reported a high *D. caninum* prevalence in necropsied Tunisian dogs (43.6%) and wild canids (29%) [21, 22], only one dog positive sample was observed in our study. The results could be explained by an underestimation of copro-helminth prevalence compared to necropsy examination, especially for cestodes, where eggs are mainly intermittently released [28]. Similar results were observed in Nigeria where necropsy of dogs reported a contamination rate of 75% for *D. caninum* [36], whereas the direct analysis of faeces revealed a prevalence ranging from 5.7 to 12.3% [37].

*Toxocara* spp. are the most common parasites living in the intestines of dogs and wild canids worldwide [38]. Humans are accidental hosts who may become infected by ingesting embryonated eggs through contaminated vegetables/water or by direct contact with dogs [10, 39]. Human infection may cause severe damage usually involving the back of the eye, the liver, and the lungs [40]. In Tunisia, information about human toxocariasis seroprevalence is nowadays absent because only case reports have been described in the literature [41–46]. In the present study, an important environmental contamination with *Toxocara* spp. eggs has been demonstrated for dog (all regions) and wild canid (Djerba and Zarzis) faeces, 27.2 and 16.3% respectively. When infected with *T. canis*, one dog could expel thousands of eggs each day. These eggs are extremely resistant in the environment and could remain infective for 5 years under favorable conditions [47]. Thus, since the environment is seriously contaminated, this zoonosis could result in an important human health problem.

Despite efforts to control the disease, cystic echinococcosis remains a serious public health problem in Tunisia [48, 49]. The life-cycle of *E. granulosus* uses domestic dogs or wild canids as the final host and ungulates as intermediate host. Humans are accidental intermediate hosts and parasite infection from dog to humans may occur, directly by contact with pets or indirectly through contaminated food or soil. In the present study, the occurrence of *E. granulosus* eggs varied according to the area but no region free of eggs was found. Despite the description of *E. granulosus* tapeworm in red foxes [21] and golden jackals [22] from north-western and central regions of Tunisia, no *E. granulosus* egg was detected in our wild canid samples although dog faeces contamination was not negligible in the same area (22.6%). The absence of *E. granulosus* in wild canids might be explained by their lack of opportunity to consume contaminated carcasses of intermediate hosts. However, in the presence of the suitable intermediate hosts in the diets of these animals, *E. granulosus* was detected in wolves but not in red foxes in Portugal [13, 50]. Red foxes are rarely infected with *E. granulosus*, usually with low parasite burdens, and are generally considered of limited importance for cystic echinococcosis transmission [47]. Therefore, it can be assumed that in Tunisia, wild canids are not implied in the disease transmission and that dogs remain the main definitive host in the life-cycle of *E. granulosus*.

The significant and positive correlation between *E. granulosus* and *Toxocara* spp. eggs observed in the present study, even in arid and desertic environment, is due to their thick outer shell (protein coat) which confers them a resistance to desiccation and to high temperature (30–80 °C) [51–53].

*Trichuris* spp*.* is found in domestic and wild canids worldwide. Although it is generally considered as non-zoonotic [54], some human cases of visceral larva migrans due to canid *Trichuris* species have been described [55–57]. Human infection is due to the accidental ingestion of embryonated eggs. *Trichuris* spp. was more geographically restricted than the other parasites found in this study and the prevalence of this parasite varied considerably (0–52.8%) from one region to another in function of the climate. Thus, in the sub-humid area (Kef), the environment is seriously contaminated (52.8%) whereas in the semi-arid neighbouring region (Kasserine) the contamination index was very low (0.7%). In the desertic and arid regions (Tataouine, Douz and Metlaoui) this parasite was totally absent. A previous study conducted in the Tunisian sub-humid region, reported similar result with a prevalence of 33% for *T. vulpis* in wild canids [22]. Precipitation and soil humidity are required for maintaining the parasite viability and *Trichuris* spp. eggs are less likely to survive in drier and sunnier locations and are unlikely to embryonate [52].

*Capillaria* spp*.* has a direct life-cycle that requires only one host. Adult worms invade the lungs of domestic and wild canids but may also be found in other mammals including humans [47]. *Capillaria* spp. was the second parasite most frequently encountered in the wild canid faeces (9.6%) and had a limited presence in dog faeces (0.1%). Similar results were described in Europe with an occurrence of 0–0.7% and 60.3–93.8% for dogs and foxes, respectively [58–60]. Thus, *Capillaria* spp. seems to be restricted to wildlife due to host-parasite interactions and dietary habits. In Tunisia, it can be assumed that wild canids act as a reservoir for capillariasis and could be responsible for the transmission of *Capillaria* populations to dogs.

## Conclusion

To our knowledge, the results of this study highlight, for the first time in Tunisia, the environmental contamination by numerous gastrointestinal parasites. In Tunisia, people’s awareness regarding zoonotic diseases transmitted by dogs is insufficient. The transmission of gastrointestinal helminths is enhanced by a high stray or semi-stray dog population, inadequate deworming treatment, the close contact of untreated dogs with humans and the favourable climatic conditions for the survival of infective stages outside the hosts. Thus, efforts should be made to increase the awareness of the presence of parasites in the environment and to implement a targeted educational program especially for the dog owners.

## Abbreviations

CI: Confidence interval
PCA: Principal components analysis
